# Automated Assessment of Thoracic-Abdominal Asynchrony in Patients with Morquio Syndrome

**DOI:** 10.3390/diagnostics11050880

**Published:** 2021-05-15

**Authors:** Madhavi V. Ratnagiri, Yan Zhu, Tariq Rahman, Mary Theroux, Shunji Tomatsu, Thomas H. Shaffer

**Affiliations:** 1 Department of Health Sciences, University of Delaware, Newark, DE 19716, USA; madhavi.ratnagiri@gmail.com; 2 Nemours Biomedical Research, Nemours/Alfred I. duPont Hospital for Children, Wilmington, DE 19803, USA; yanzhu2886@gmail.com (Y.Z.); tariq.rahman@nemours.org (T.R.); shunji.tomatsu@nemours.org (S.T.); 3 Department of Anesthesiology and Perioperative Medicine & Nemours Biomedical Research, Nemours/Alfred I. duPont Hospital for Children, Wilmington, DE 19803, USA; mary.theroux@nemours.org; 4 Department of Pediatrics, Gifu University, Gifu 501-1193, Japan; 5 Department of Pediatrics, Sidney Kimmel Medical College at Thomas Jefferson University, Philadelphia, PA 19107, USA; 6 Center for Pediatric Lung Research, Nemours/Alfred I. duPont Hospital for Children, Wilmington, DE 19803, USA; 7 Department of Physiology and Pediatrics, Lewis Katz School of Medicine at Temple University, Philadelphia, PA 19140, USA

**Keywords:** automated pulmonary assessment, Morquio syndrome, noninvasive pulmonary diagnostics, thoracic-abdominal asynchrony

## Abstract

Morquio syndrome is a rare disease caused by a disorder in the storage of mucopolysaccharides that affects multiple organs, including musculoskeletal, respiratory, cardiovascular, and digestive systems. Respiratory failure is one of the leading causes of mortality in Morquio patients; thus, respiratory function testing is vital to the management of the disease. An automated respiratory assessment methodology using the *pneu*RIP device and a machine-learning algorithm was developed. *pneu*RIP is a noninvasive approach that uses differences between thoracic and abdominal movements (thoracic-abdominal asynchrony) during respiration to assess respiratory status. The technique was evaluated on 17 patients with Morquio (9 females and 8 males) between the ages of 2 and 57 years. The results of the automated technique agreed with the clinical assessment in 16 out of the 17 patients. It was found that the inverse cumulative percentage representation of the time delay between the thorax and abdomen was the most critical variable for accurate evaluation. It was demonstrated that the technique could be successfully used on patients with Morquio who have difficulty breathing with 100% compliance. This technique is highly accurate, portable, noninvasive, and easy to administer, making it suitable for a variety of settings, such as outpatient clinics, at home, and emergency rooms.

## 1. Introduction

Morquio syndrome (technically termed “mucopolysaccharidosis type IV [MPS IV]”) is a rare disorder that occurs in 1 out of 200,000 to 300,000 births [1,2,3]. Individuals with this disorder have a deficiency of a lysosomal enzyme, either N-acetylgalactosamine-6-sulfate sulfatase (GALNS; Morquio A) or *β*-galactosidase (Morquio B) and cannot break down specific glycosaminoglycans (GAGs). This enzyme deficiency results in the accumulation of GAGs, keratan sulfate (KS), and/or chondroitin-6-sulfate (C6S) within various tissues, especially bone, cartilage, and connective tissues, causing progressive damage to multiple organs. In general, type A comprises the more severe form.

The disease usually manifests between about 1 and 3 years of age with symptoms such as kyphosis (hump-back), short neck, cervical spinal cord compression, protrusion of the chest bone (pectus carinatum), short stature, abnormal waddling gait, or knock knee (genu valgum), or a combination of these. Patients with MPS IV typically require frequent orthopedic surgeries of the spine (cervical decompression/fusion), hips (reconstruction and replacement), legs (osteotomy), knees (8-plate), etc. Cervical decompression/fusion surgery is required to stabilize their cervical region, prevent spinal compression, and avoid paraplegia and possible death. Another common cause of death is respiratory problems including progressive airway narrowing, which is life-threatening with age. An imbalance of growth, including short neck and stature, adenotonsillar hypertrophy, large mandible, or pectus carinatum, causes respiratory distress and makes intubation and extubation difficult, driving challenges in the anesthetic process during surgeries [4].

Standard clinical respiratory measures, such as spirometry, require active effort and cooperation from patients. Consequently, these tests provide low compliance rates among children and those with disabilities [5,6]. Thus, proper respiratory assessment with minimal voluntary effort is a critical need. Furthermore, these conventional techniques involve collection of expired gases, which may contain infectious viruses and require careful cleaning techniques. With the recent events due to COVID-19, these issues have drawn further attention. 

In the proposed method, air flow, respiratory rate (RR), tidal volume (Vt), end-tidal CO_2_ (ETCO_2_), heart rate (HR), and pulse oximetry measurements can be obtained by an integrated pneumotach (PNT) system for simultaneous, real-time, noninvasive measurements.

Respiratory inductance plethysmography (RIP) is a noninvasive method that can be used to assess respiratory function by recording the rib cage (RC) and abdominal (ABD) movements during respiration [7,8]. One hundred percent compliance with the RIP noninvasive test was obtained in pilot studies on patients with Morquio syndrome [9], pediatric patients in the emergency room admitted for asthma [10], infants with bronchopulmonary dysplasia [11], and neuromuscular patients [12,13].

Respiratory inductance plethysmography assesses respiratory function by measuring differences in amplitude and synchrony between the thoracic and abdominal movements during tidal breathing. For healthy individuals, the time delay is near zero because as a person breathes the motions of the thoracic and abdominal compartments are almost synchronous for efficient gas exchange. The lag between thoracic and abdominal movements is known as thoracic-abdominal asynchrony (TAA), indicating the severity of pulmonary dysfunction. Other parameters such as percentage RC (%RC), which is an indication of the RC contribution to Vt, and the labored breathing index (LBI), which is a measure of additional respiratory effort due to asynchronous breathing, are other indices computed to assess TAA [14,15].

Thoracic-abdominal asynchrony measured using RIP has been identified as a clinical diagnostic for many pulmonary diseases such as asthma [10], chronic obstructive pulmonary disease [16], and neuromuscular disorders and other diseases such as heart failure and panic disorder [17]. Data from Giordano et al. [10] on pediatric patients in the emergency department showed that tidal breathing tests measured by the RIP device could provide objective measures of the severity of asthma. Many studies [18,19,20,21] have found measurements from RC and ABD wall movements to be valuable predictors of obstructive sleep apnea. Multiple studies [9,22] conducted specifically on patients with Morquio syndrome compared various pulmonary function testing methods, such as spirometry, impulse oscillometry system, pneumatography, and RIP, and demonstrated that RIP is a reliable indicator of respiratory function.

The standard RIP device (Respitrace System, Sensormedics, Yorba Linda, CA, USA) requires post-processing to assess respiratory function, thus requiring additional effort by the physician, making it unsuitable for real-time monitoring. In contrast, *pneu*RIP, a recently developed device, records RC and ABD movements similar to the standard RIP but transmits the data wirelessly to an iPad that also calculates and displays the respiratory function parameters in real time [13,14]. Similarly to the standard RIP, the *pneu*RIP records the movement of the thorax and abdomen using inductive wires embedded in bands that are placed around the chest and stomach. It then uses fast Fourier transforms to measure indices such as the phase difference (ϕ) between the signals, which effectively is the time delay between the thoracic and abdominal movements. The automated approach to computing the respiratory parameters reduces the extra effort for physicians in manually calculating the time delay or merely inferring the TAA by visual inspection of signals.

Using the indices computed by the *pneu*RIP, significant differences between healthy and neuromuscular patients were observed via ANCOVA analysis [13]. However, these analyses used mean values and did not consider the variation over time. If there are changes in the parameters over time and their distribution could be uniform, bimodal, or have extreme outliers [13], then the mean estimate will be imprecise. Accurate interpretation of the breathing patterns, by observing their variability over time and without outlier bias, is crucial for predicting impending respiratory dysfunction and failure. Hence, a median-based robust statistic that uses a cumulative frequency metric, specifically the inverse cumulative percentage (ICP), was developed to assess variation over time [15]. A novel visual representation of ICP identifies the median statistic and provides relative durations of normal and abnormal breathing in the patient. Pulmonologists found the graphic display of ICP very useful, intuitive, and easy to interpret [15].

Further, using the ICP parameter, an accurate automated machine learning (ML) system for the classification of normal and abnormal breathing was developed and published [15]. The model was trained using an elastic-net regularization algorithm and with signals recorded from healthy pediatric volunteers while they were breathing normally and with a resistive load to simulate abnormal breathing. It was shown that the model assessed pulmonary dysfunction among children with neuromuscular disorders very successfully and was consistent with expert pulmonologists’ evaluations [15]. Because people with Morquio syndrome have small stature with lung capacities similar to those of children, the same ML model to evaluate respiratory function among subjects with Morquio syndrome was used.

## 2. Materials and Methods

### 2.1. Data Acquisition and Protocol

This study was approved by the Institutional Review Board (IRB) at Nemours/Alfred I. duPont Hospital for Children, and assent/consent from the subjects and subjects’ guardians was obtained. All subjects were seated comfortably in a chair/wheelchair and asked to remain still for the recording duration. A recording session lasted about 3 min.

Each subject’s cardiopulmonary vital signs (air flow, Vt, ETCO_2_, HR, and oxygen saturation [SPO_2_]) were acquired using an integrated PNT system (CO2SMO; Novametrix Medical Systems, Wallingford, CT, USA) for simultaneous, real-time noninvasive measurements [9]. The CO2SMO Pulse Oximeter [9] measured oxygen saturation levels in the blood; this is an indirect measure of respiratory function and does not require arterial blood sampling. The device has a small sensor clipped to the subject’s index finger to measure HR and blood SPO_2_.

**Figure 1:**
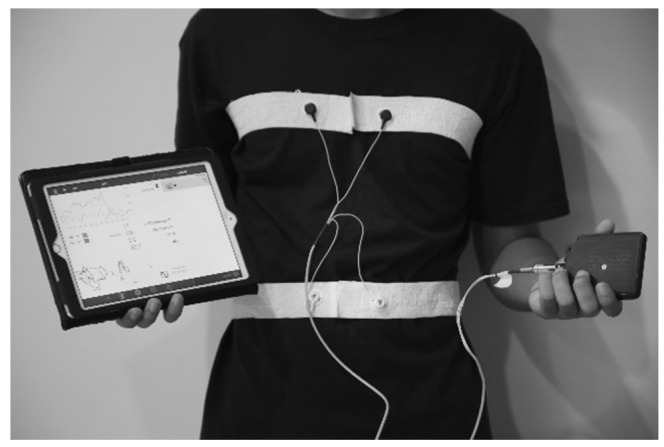
The *pneu*RIP device with elastic bands strapped around the RC and ABD of a subject. The bands are connected to a box that transmits data to the iPad.

The *pneu*RIP method measures changes in the cross-sectional area of the RC and the ABD during breathing. It consists of two elastic bands, each with an embedded insulated wire placed around the RC and the ABD forming a coil (Figure 1). A small AC current passing through the coil generates a self-inductance that oscillates in a cyclic pattern that tracks changes in the cross-sectional area associated with respiration. The inductive signals are transmitted wirelessly to the iPad (Apple Inc., Cupertino, CA, USA) (Figure 1), where the various TAA indices are calculated and displayed. Data were collected for 3 min from each subject. Raw signals and the Konno–Mead loops [7] were monitored during the recording to validate good signal quality.

Data were recorded from 17 subjects with Type A Morquio syndrome. They were between the ages of 2 and 57 years with an average height of 102.5 cm and a wide range of clinical severity. Subjects were recruited during a Morquio symposium organized at Nemours/Alfred I. duPont Hospital for Children. To develop an ML model, a training set of *pneu*RIP recordings was needed. Since Morquio patients have small statures and lung capacities comparable with those of children, recordings from healthy children were chosen to train the model. The training data set included recordings from 10 typically developing subjects (10–17 years) under two different breathing conditions. One was quiet tidal breathing and the other was with an added resistive load. The resistive load was added to simulate asynchronous breathing encountered during respiratory dysfunction. Resistance was provided by an external bidirectional laminar resistive load (Hans Rudolph, Shawnee, KS, USA) 20 cm H_2_O/L/sec, which was placed in the mouth while the subject wore a nose-clip. The resistive load was used because it was easier to administer on children. Allen et al. [11] showed that changes in the relative movements of RC and ABD are nonspecific to whether there is obstructive (as experienced by the subjects breathing through the resistive load) or restrictive lung problems.

### 2.2. Computation of the Respiratory Indices

To assess respiratory function and TAA, multiple indices such as RR, ETCO_2_, SPO_2_, %RC, LBI, and phase difference (ϕ) were measured. The RR, ETCO_2_, and SPO_2_ were measured using a pulse oximeter, whereas %RC, LBI, and ϕ were calculated from the recordings of the RC and ABD movements by the *pneu*RIP.

Respiratory rate is the number of breaths per minute, SPO_2_ is the level of oxygen saturation in the blood, and ETCO_2_ is the amount of carbon dioxide in the exhaled air. The CO2SMO monitor provides all these measurements.

**Figure 2:**
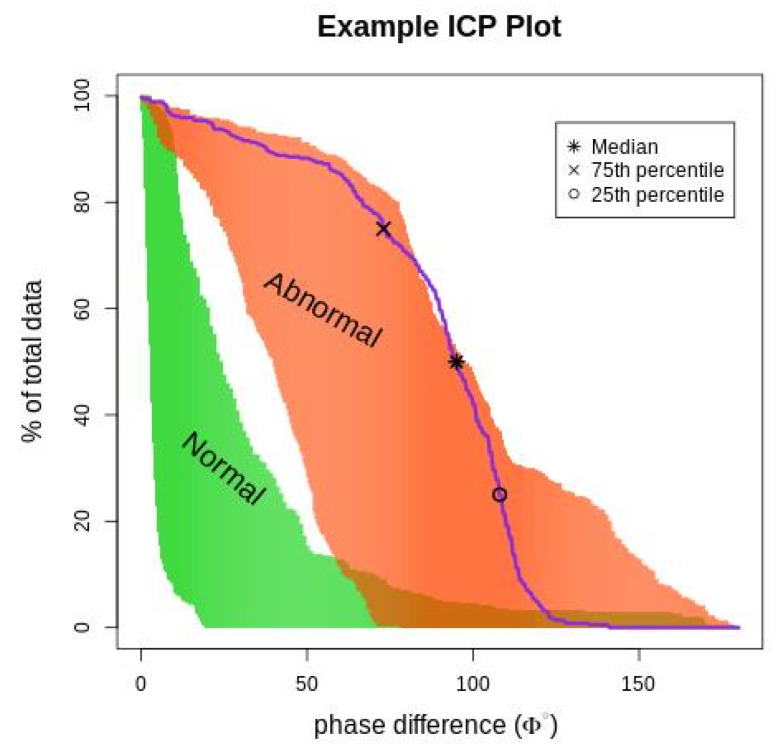
The figure depicts an ICP phase plot (purple line) for a subject superimposed on normal and abnormal breathing areas. The (*) represents the median phase difference value, (**x**) represents the 75th percentile value, and (**o**) represents the 25th percentile. The orange (abnormal) area is the ICP value for subjects breathing asynchronously because of the resistive load, and the green (normal) area is the ICP value for healthy subjects breathing normally.

Phase difference (ϕ) is an essential index for identifying TAA; it is the time delay between RC and ABD movements (measured in degrees). The phase over the 3 min duration was characterized as an ICP to represent the frequency of occurrence of each phase angle within the data recorded. The plot of ICP is shown in Figure 2; the normal and abnormal regions were obtained by plotting the ICP values of the training data. The data from healthy subjects are indicated by green, and the data from healthy subjects breathing through the resistive load are indicated by orange.

The %RC is a ratio of the absolute amplitude of rib cage signal to the sum of the absolute amplitudes of the rib cage and abdomen signals. The %RC is an indication of RC contribution to Vt and is presented as a percent of the combined RC and ABD movements. The average %RC in normal breathing is close to 50%, indicating that the RC and the ABD contribute about the same amount to the respiratory Vt [23].

The LBI is a measure of additional respiratory work effort due to asynchronous breathing [13,14,15]. The LBI is a ratio that indicates the efficiency of breathing. The numerator is the “maximum compartmental volume” and is calculated as the sum of the absolute values of the RC and ABD compartments if they moved in perfect unison. The denominator is the Vt, which is the sum of the absolute values of the RC and ABD compartments as recorded. If RC and ABD signals are in phase (representing synchronous breathing), then numerator and denominator terms will be similar, and the LBI would be 1.

### 2.3. ML Model Training and Classification

An ML model for the automated identification of normal and abnormal TAA patterns was utilized for patients with Morquio [15]. A logistic regression method called elastic-net regularization was used to train the ML model. This classification technique was chosen because of the small sample size, which limits the complexity of the algorithm. However, a logistic regression algorithm does have high bias and low variance, which was offset by the use of elastic-net regularization. The ‘glmnet’ package in R [24] was used to run the elastic-net training. The ICP values were considered as the predictors, and the classification into normal or abnormal was the response variable as shown in Equation (1) below. The model prediction (*ŷ*) of normal or abnormal breathing was calculated using a weighted sum of the predictor variables (*x_p_*) as shown in (1).

(1) $$\hat{y}={\hat{\beta }}_{0}+x_{1}{\hat{\beta }}_{1}+\cdots +x_{p}{\hat{\beta }}_{p}$$

**Figure 3:**
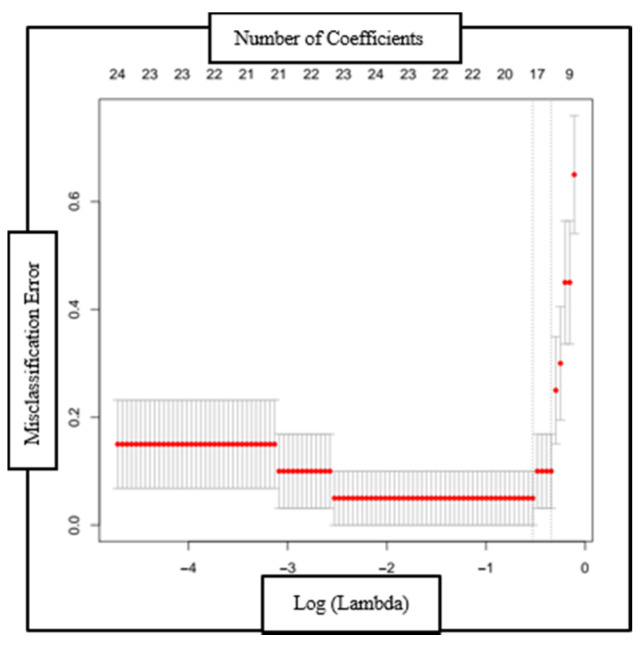
Plot of the misclassification error as a function of log (Lambda) and the reduction in coefficients (top axis) with each validation run. The red dotted line is the region one standard deviation from the minimum classification error point.

The optimal bias-variance trade-off was determined empirically using a 10-fold cross validation run (Figure 3). The model’s performance was evaluated by comparison with an expert’s judgment. Pulmonary experts evaluated and compared RIP-time measures with the ICP plots and categorized them as normal or abnormal TAA patterns, and the model’s classification was compared with the experts’ categorization. In addition, the model’s classification was also compared with an expert’s opinion of the respiratory function based on clinical assessment.

## 3. Results

### 3.1. Respiratory Parameters

The values of the respiratory indices from each patient with Morquio were compared with those of the healthy individuals. The range of the respiratory index values for healthy individuals with respect to age was obtained by Balasubramaniam et al. [23] through an extensive literature review. Respiratory index values were also considered as a function of height, and no correlation was observed.

#### 3.1.1. Respiratory Rate

**Figure 4:**
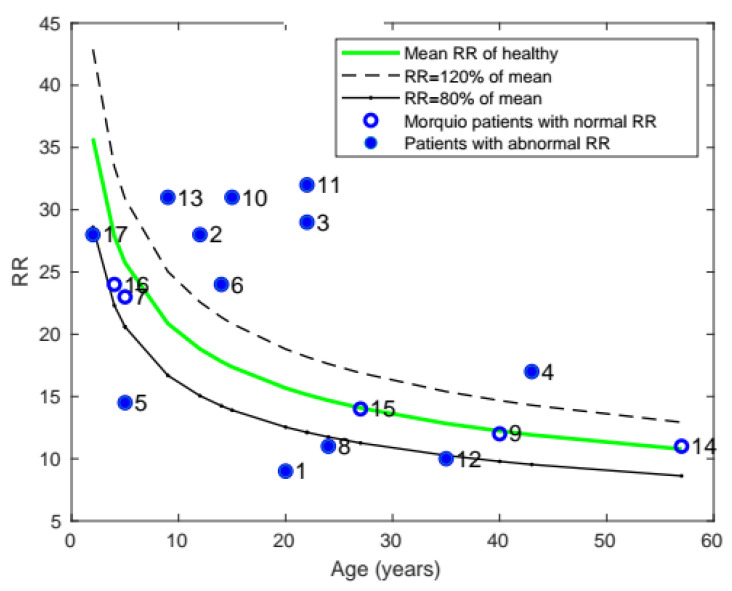
Plot of mean RR with age, comparing the RR of patients with Morquio with that of healthy individuals of the same age (the data points represent subject numbers).

As noted in Figure 4, the RR of patients with Morquio was compared with that of healthy individuals of the same age. As can be seen from the plot, 12 of the 17 patients with Morquio had RR greater than or lower than 20% of the average RR of the healthy mean.

#### 3.1.2. Blood Oxygen Levels (SPO_2_)

All patients were found to have normal (>95%) SPO_2_ levels.

#### 3.1.3. End-Tidal Carbon Dioxide (ETCO_2_)

**Figure 5:**
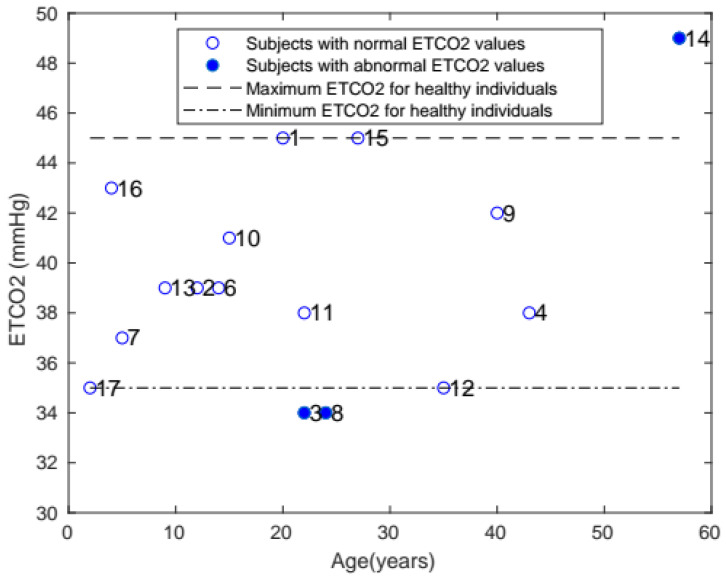
Plot of ETCO_2_ with age, comparing the ETCO_2_ of patients with Morquio with that of healthy individuals of the same age (the data points represent subject numbers).

The ETCO_2_ of each patient is shown in Figure 5. The healthy range for ETCO_2_ was 35–45 mmHg, and only 3 patients with Morquio had abnormal levels outside that range.

#### 3.1.4. Percentage Rib Cage (%RC)

**Figure 6:**
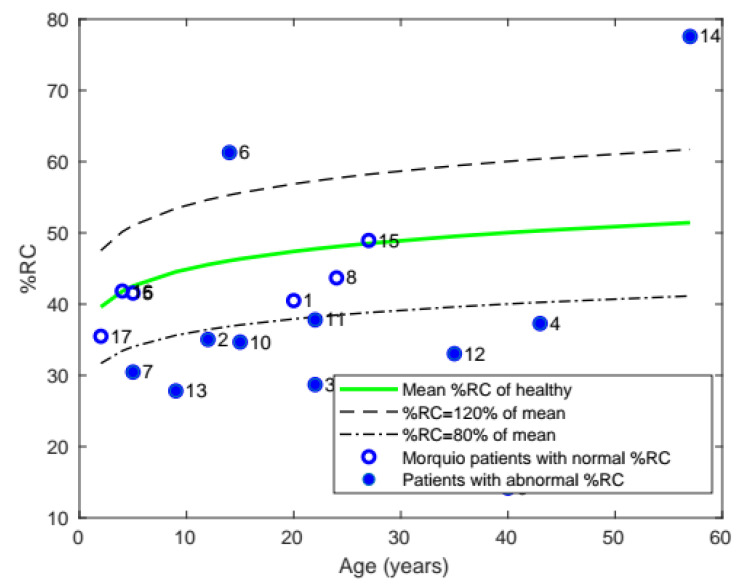
Plot of mean %RC with age, comparing the %RC of patients with Morquio with that of healthy individuals of the same age (the data points represent subject numbers).

Similarly, the average %RC of patients with Morquio was compared with that of healthy individuals of the same age (Figure 6). The majority of the patients (10 of the 17) were found to have abnormal %RC.

#### 3.1.5. Labored Breathing Index

**Figure 7:**
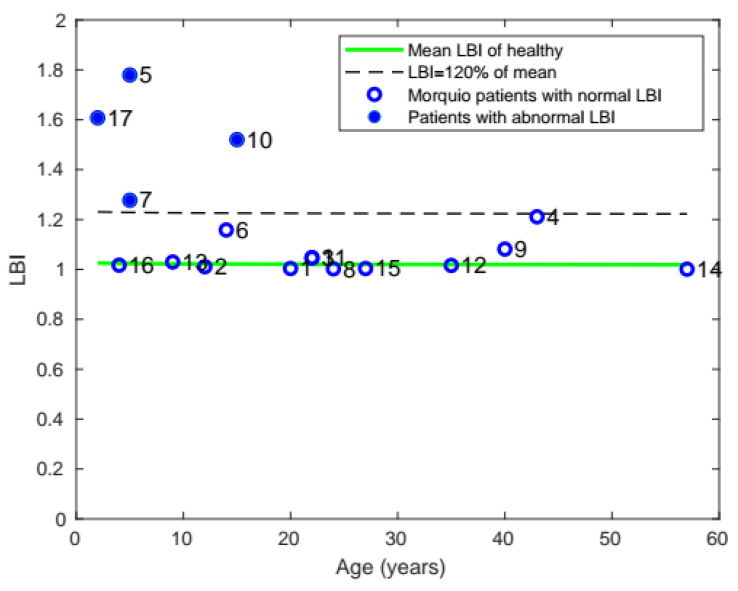
Plot of mean LBI with age, comparing the LBI of patients with Morquio with that of healthy individuals of the same age (the data points represent subject numbers).

Comparing the LBI of patients with Morquio with that of healthy individuals, it was found that a total of 6 patients with Morquio had LBI values out of the normal range and 4 patients had LBI greater than 20% of the mean value (Figure 7).

#### 3.1.6. Phase Difference

**Figure 8:**
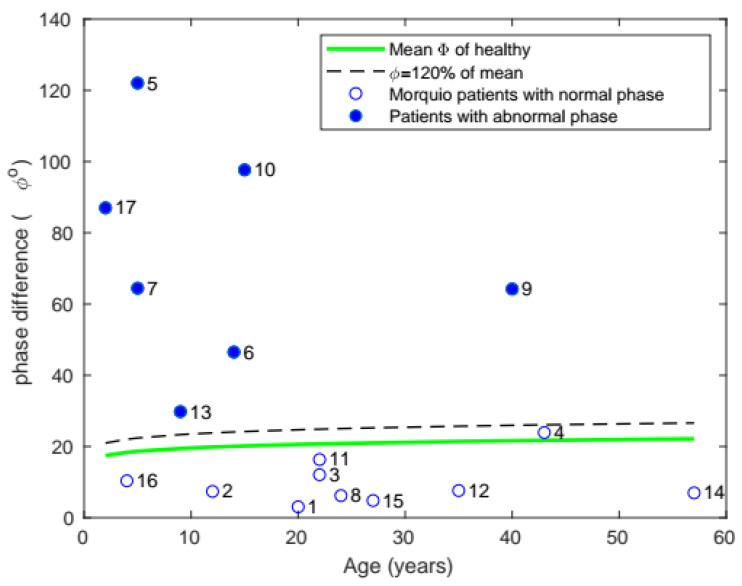
Plot of mean phase with age, comparing the phase of patients with Morquio with that of healthy individuals of the same age (the data points represent subject numbers).

In mean phase difference (Figure 8), 7 of the 17 subjects were found to have phase differences above 20% of the healthy population’s mean.

#### 3.1.7. Inverse Cumulative Phase

Instead of looking at just the mean phase, the ICP was computed, and ML modelling was used to identify abnormal vs. normal breathing among the patients with Morquio (Table 1).

### 3.2. Model Predictions

The elastic-net model’s performance was determined by comparing the model’s response with the expert’s evaluation [15]. One hundred percent accuracy was obtained as the model’s prediction exactly matched the human expert’s judgment. Recall that the models were trained on abnormal breathing patterns generated by healthy subjects breathing through a resistive load to simulate TAA breathing. At the same time, the assessment set included recordings of patients with Morquio. Yet, the model predictions on the abnormal breathing patterns collected from patients with Morquio were excellent. This indicates that the method used to simulate TAA breathing was a good representation of the breathing pattern of patients with Morquio while in respiratory distress and that the training algorithm was effective in generalizing to a data set from a different population.

### 3.3. Clinical Diagnosis and Surgeries

A pediatric anesthesiologist (MT) provided clinical assessment of the respiratory function of each of the patients with Morquio (Table 2). Any surgeries related to the neck or trachea were also noted to observe if that might have an effect on their respiration. Tracheal narrowing [25] and airway obstruction [26] are known symptoms of patients with Morquio A syndrome. Tracheal reconstruction surgery [27], which includes tracheal and vascular reconstruction, is done to relieve tracheal obstruction and also addresses lower airway obstruction. Pizzaro et al. [27] showed that this surgery resolved a 16-year-old patient’s respiratory symptoms. Please note that the clinical assessment was given prior to the tracheal reconstruction surgery.

A summary of all the respiratory function variables that were measured is presented in Table 3; SPO_2_ is not included since all patients had normal levels. A column for surgery indicates if the patient had neck/tracheal surgery. The column for clinical diagnosis indicates if the anesthesiologist’s assessment of the patients’ respiratory function was normal or abnormal.

Table 3 shows that there were four patients with abnormal ICP (✕ in column 8), and three of those four patients were diagnosed as having respiratory problems (✕ in column 10); information was unavailable for one of them (subject #7). In addition, there were six other patients who were diagnosed clinically as having respiratory trouble who did not have abnormal ICP values, and all had tracheal or neck fusion surgeries. It is noteworthy that all patients who had tracheal/neck surgeries had normal ICP phase.

## 4. Discussion

There are multiple diagnostic methods available to assess respiratory function; some are invasive and hard to administer, such as spirometry, and others, such as sleep apnea tests, are noninvasive but require technicians to administer. However, *pneu*RIP is noninvasive, needs minimal expertise to administer, and requires just about 5 min of the subject’s time but can also be used for real-time monitoring. One hundred percent compliance has been shown in all studies [13,14,15], and, furthermore, both restrictive and obstructive disorders can be identified with this method [12]. In addition, diagnosing respiratory dysfunction has been automated using ML modeling [15], making it adaptable to in-home or any hospital setting, such as the emergency department.

From the results summarized in Table 3, even though all patients had normal blood oxygen levels, 12 of them had abnormal respiratory rates, and 3 had abnormal ETCO_2_ levels. During pulmonary testing, most patients become over conscious of their breathing, and, sometimes, abnormal RR rates are associated with the emotional aspect of the measurement process. Of the three patients who had abnormal ETCO_2_ values, the patient with high ETCO_2_ was 57 years old and a long-time smoker (#14); this result is consistent with high CO_2_ levels and chronic obstructive lung disease. The testing process, as noted above, suggests the reason why the two other patients (#3 and #8) had low ETCO_2_ levels. In the present study, a significant correlation between RR and ETCO_2_ levels could not be found; however, this was a small data set of patients with a rare disease. The sample size was too small to establish a correlation between these parameters. A correlation between height and these respiratory parameters was also not observed, since within the sample size the range of heights was limited (97.8–105.4 cm), with most patients already at their mature height at the time of the measurements.

Of the three patients with sleep apnea problems and eight with airway obstruction, one patient (subject #3) had both obstructive sleep apnea (OSA) and airway obstruction. Thus, there were a total of 10 subjects who had OSA or airway obstruction problems or both. Only two of them were identified as having abnormal ICP, and these two were the only ones who did not undergo tracheal reconstruction surgery or a cervical fusion. The remaining eight subjects who had surgery all had normal ICP phase values. The neck/tracheal surgeries performed to stabilize the cervical region may indirectly help to alleviate respiratory stress due to TAA.

Two other subjects, #7 and #17, also had not undergone any tracheal surgeries, probably because they were young (<15 years old) and were found to have abnormal ICP. The anesthesiologist found subject #7 to have clinical indications of respiratory problems consistent with the ICP evaluation. Subject #17 was the only patient who had abnormal ICP values where the anesthesiologist found no respiratory issues. This might have been because the subject was only 2 years old, and the analysis is based on data collected from children above the age of 10 years. Prior work with RIP [23] has found that RC and ABD motions are stabilized only after 5 years. By including more data from younger age groups it is hoped that a better assessment for the younger population of patients can be provided as well.

Thus, the ML algorithm’s identification of abnormal TAA agreed with the clinical assessment in all but one patient’s respiratory function. A simple algorithm, elastic-net, was used for the automated classification task because of the small sample size, and yet the accuracy was high; only one patient’s diagnostic classification did not match the clinical assessment. The data size was small for ML training; however, it was a large data set for a rare disease that occurs in 1 in 200,000 to 300,000 births [1,2,3]. Further, since the ICP variable can be used to observe the progression of the patients’ respiratory status over time, it is ideal for monitoring and managing their respiratory function until they are old enough to have a surgery. Additionally, with the automated ML modeling, respiratory status can even be measured at home, and, if the respiratory function deteriorates, the physician can be alerted.

## 5. Conclusions

An automated, noninvasive device to evaluate respiratory function was implemented. It has been shown to be an accurate detection system that matches clinical diagnostic assessment. In addition, the informative graphical display of the ICP feature along with RIP-time measures can be used to enhance observation and progression of the respiratory status of a patient over time. Because of the noninvasive nature of the test, 100% compliance was obtained from the subjects, and, because of the ease of administering the test and the automated ML diagnostic evaluation, it can be used in any hospital or home setting.

## Figures and Tables

**Table 1:** ML model classification results based on the ICP values of 17 patients with Morquio.

	Number of Normally Breathing Subjects	Number of Abnormally Breathing Subjects	Total Subjects with Morquio
ML model identification	13	4	17

**Table 2:** Clinical assessment of the respiratory function of the patients with Morquio by an anesthesiologist and list of surgeries related to the neck and trachea. OSA, obstructive sleep apnea; RAD, restrictive airway disorder; NA, no medical information was available; “none”, no neck or tracheal surgery was performed.

Patient ID	Clinical Diagnosis	Surgery Related to Trachea and Cervical Regions
1	NA	Cervical fusion
2	Moderate narrowing of trachea	Cervical fusion, tracheal reconstruction surgery
3	OSA, RAD, restrictive lung disease	Cervical fusion, tracheal reconstruction surgery
4	NA	Cervical fusion
5	No respiratory issues	Spinal decompression
6	Severe central airway obstruction	Cervical fusion, tracheal reconstruction surgery
7	NA	NA
8	Mild central airway obstruction	Cervical fusion
9	NA	NA
10	Severe central airway obstruction	None
11	OSA, mild narrowing of central airway	Tracheal reconstruction surgery
12	Mild OSA, moderate central airway obstruction	Neck fusion
13	Severe central airway obstruction	None
14	NA	NA
15	Severe central airway obstruction	Tracheal reconstruction surgery
16	No respiratory issues	Cervical fusion
17	No respiratory issues	None

**Table 3:** Subjects with normal and abnormal values for each of the variables measured and the clinical diagnosis are shown here with ✓ representing normal and ✕ representing abnormal, “NA” representing unavailable information, and “none” meaning no surgery related to the neck or the trachea was performed on the patient.

Patient #	Age	RR	ETCO_2_	%RC	LBI	Phase	ICP (Phase)	Surgery	Clinical Diagnosis
1	20	✕	✓	✓	✓	✓	✓	✓	NA
2	12	✕	✓	✕	✓	✓	✓	✓	✕
3	22	✕	✕	✕	✓	✓	✓	✓	✕
4	43	✕	✓	✕	✓	✓	✓	✓	NA
5	5	✕	NA	✓	✕	✕	✓	✓	✓
6	14	✕	✓	✕	✓	✕	✓	✓	✕
7	5	✓	✓	✓	✕	✕	✕	NA	NA
8	24	✕	✕	✓	✓	✓	✓	✓	✕
9	40	✓	✓	✓	✓	✕	✓	NA	NA
10	15	✕	✓	✕	✕	✕	✕	None	✕
11	22	✕	✓	✕	✓	✓	✓	✓	✕
12	35	✕	✓	✕	✓	✓	✓	✓	✕
13	9	✕	✓	✕	✓	✕	✕	None	✕
14	57	✓	✕	✕	✓	✓	✓	NA	NA
15	27	✓	✓	✕	✓	✓	✓	✓	✕
16	4	✓	✓	✓	✓	✓	✓	✓	✓
17	2	✕	✓	✓	✕	✕	✕	None	✕

## Data Availability

All relevant data are within the manuscript and its Supporting Information files.
